# eNAMPT is a novel therapeutic target for mitigation of coronary microvascular disease in type 2 diabetes

**DOI:** 10.1007/s00125-024-06201-9

**Published:** 2024-06-19

**Authors:** Lei Gao, Francisco J. Ramirez, Jody Tori O. Cabrera, Mathews V. Varghese, Makiko Watanabe, Atsumi Tsuji-Hosokawa, Qiuyu Zheng, Mingya Yang, Md Rahatullah Razan, Carrie L. Kempf, Sara M. Camp, Jian Wang, Joe G. N. Garcia, Ayako Makino

**Affiliations:** 1 Department of Medicine, University of California, San Diego, La Jolla, CA USA; 2 Center for Inflammation Science and Systems Medicine, The Herbert Wertheim UF Scripps Institute for Biomedical Innovation & Technology, University of Florida, Jupiter, FL USA; 3 Department of Physiology, The University of Arizona, Tucson, AZ USA; 4 Department of Pulmonary and Critical Care Medicine, The Second Affiliated Hospital of Guangzhou Medical University, Guangzhou, China; 5 State Key Laboratory of Respiratory Disease, Guangzhou Institute of Respiratory Disease, The First Affiliated Hospital of Guangzhou Medical University, Guangzhou, China

**Keywords:** Coronary blood flow, Endothelial cell function, Microvascular complications, PBEF, Type 2 diabetes, Visfatin

## Abstract

**Aims/hypothesis:**

Individuals with diabetes are at high risk of cardiovascular complications, which significantly increase morbidity/mortality. Coronary microvascular disease (CMD) is recognised as a critical contributor to the increased cardiac mortality observed in people with diabetes. Therefore, there is an urgent need for treatments that are specific to CMD. eNAMPT (extracellular nicotinamide phosphoribosyltransferase) is a damage-associated molecular pattern and TLR4 ligand, whose plasma levels are elevated in people with diabetes. This study was thus designed to investigate the pathogenic role of intracellular nicotinamide phosphoribosyltransferase (iNAMPT) and eNAMPT in promoting the development of CMD in a preclinical murine model of type 2 diabetes.

**Methods:**

An inducible type 2 diabetic mouse model was generated by a single injection of low-dose streptozocin (75 mg/kg, i.p.) combined with a high-fat diet for 16 weeks. The in vivo effects of i/eNAMPT inhibition on cardiac endothelial cell (CEC) function were evaluated by using *Nampt*^+/−^ heterozygous mice, chronic administration of eNAMPT-neutralising monoclonal antibody (mAb) or use of an NAMPT enzymatic inhibitor (FK866).

**Results:**

As expected, diabetic wild-type mice exhibited significantly lower coronary flow velocity reserve (CFVR), a determinant of coronary microvascular function, compared with control wild-type mice. eNAMPT plasma levels or expression in CECs were significantly greater in diabetic mice than in control mice. Furthermore, in comparison with diabetic wild-type mice, diabetic *Nampt*^+/−^ heterozygous mice showed markedly improved CFVR, accompanied by increased left ventricular capillary density and augmented endothelium-dependent relaxation (EDR) in the coronary artery. NAMPT inhibition by FK866 or an eNAMPT-neutralising mAb significantly increased CFVR in diabetic mice. Furthermore, administration of the eNAMPT mAb upregulated expression of angiogenesis- and EDR-related genes in CECs from diabetic mice. Treatment with either eNAMPT or NAD^+^ significantly decreased CEC migration and reduced EDR in coronary arteries, partly linked to increased production of mitochondrial reactive oxygen species.

**Conclusions/interpretation:**

These data indicate that increased i/eNAMPT expression contributes to the development of diabetic coronary microvascular dysfunction, and provide compelling support for eNAMPT inhibition as a novel and effective therapeutic strategy for CMD in diabetes.

**Graphical Abstract:**

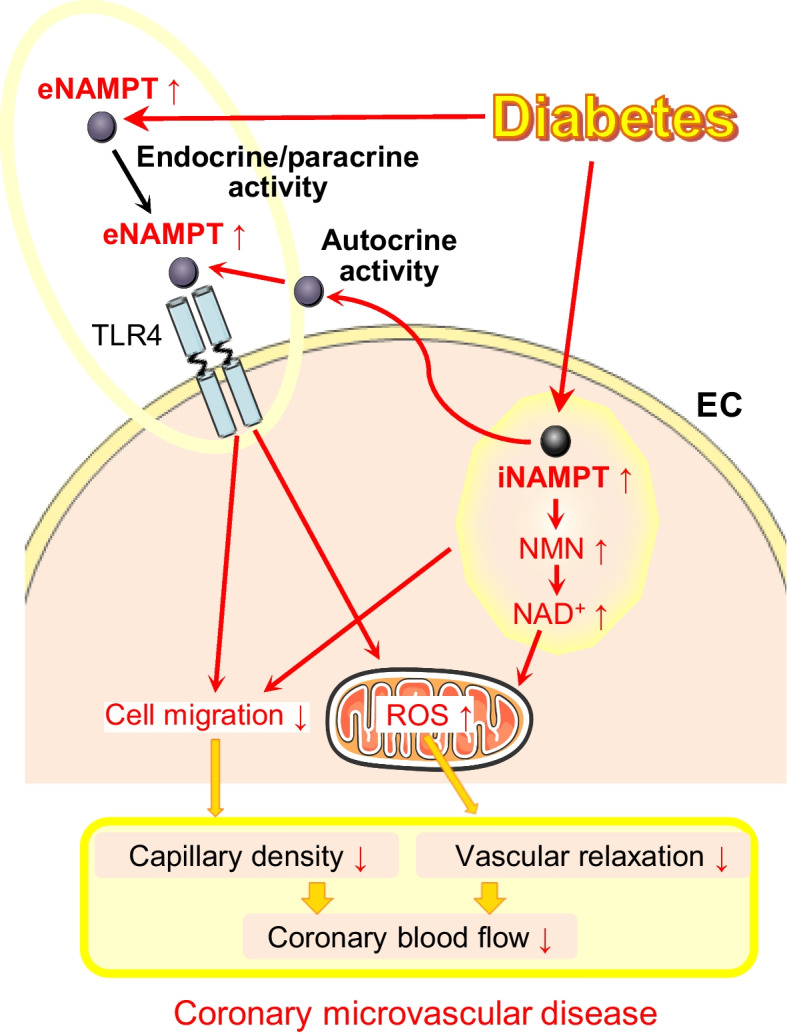

**Supplementary Information:**

The online version of this article (10.1007/s00125-024-06201-9) contains peer-reviewed but unedited supplementary material.


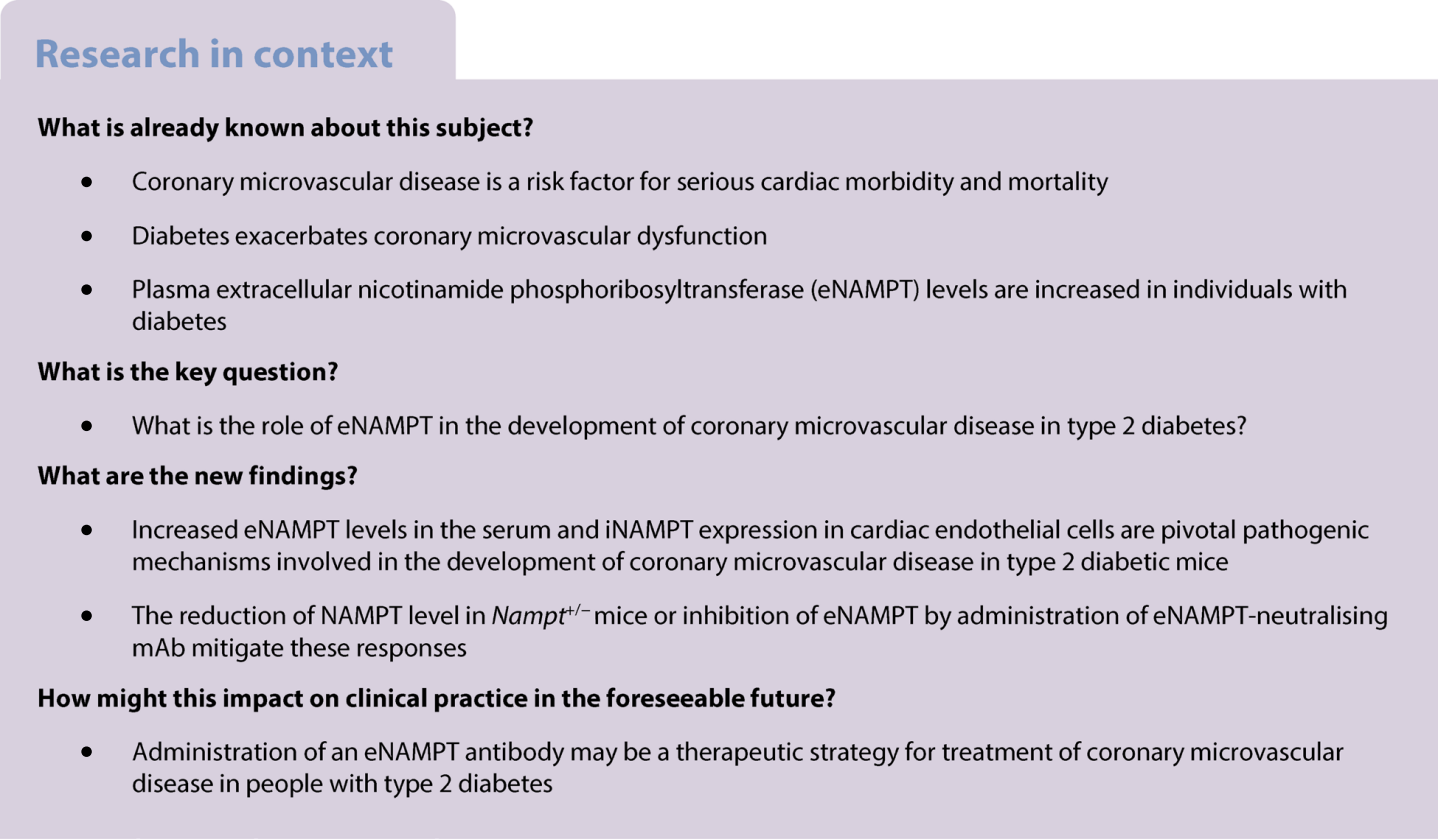


## Introduction

Diabetes, the 7th leading cause of death globally due to the comorbid conditions of diabetic cardiomyopathy, obstructive coronary artery disease (CAD), coronary microvascular disease (CMD) or stroke, affects over 37 million people in the USA. CMD is now recognised as a key risk factor for cardiac ischaemia and heart attack [1, 2]. Coronary flow reserve is a well-accepted assessment of coronary microvascular function in patients with CMD [3], and reduced coronary flow velocity reserve (CFVR) is observed in both diabetic patients and preclinical models of diabetes [4–6]. Importantly, diabetic patients with CMD exhibit higher cardiac mortality than non-diabetic patients with CMD or patients with diabetes but without CMD [5]. Current efforts to treat CMD remain reliant on the use of CAD medications, mandating further studies to understand CMD pathogenic mechanisms to develop novel CMD therapeutic approaches in people with diabetes.

The mechanisms responsible for reduced CFVR include microvascular rarefaction, increased vasospasm, attenuated small coronary artery vasodilation and remodelling [2, 7, 8]. Vascular endothelial cells (ECs) play significant roles in revascularisation, regulation of vascular tone and maintenance of anticoagulant level and barrier integrity/function. We and others have shown that capillary density in the heart is decreased in diabetic individuals and animal models of diabetes [6, 9], and that endothelium-dependent relaxation (EDR) in coronary arteries (CAs) is attenuated in animal models of diabetes due to reduced NO bioavailability [10, 11] and impaired endothelium-dependent hyperpolarisation (EDH)-mediated vasodilatation [6]. Therefore, restoring coronary endothelial function in diabetes would improve coronary microvascular function and decrease cardiac mortality in diabetic individuals.

Nicotinamide phosphoribosyltransferase (NAMPT), also known as pre-B cell colony-enhancing factor (PBEF) or visfatin, is encoded by the *Nampt* gene on human chromosome 7 and mouse chromosome 12, and is expressed in every organ and cell type. Intracellular NAMPT (iNAMPT) and extracellular NAMPT (eNAMPT) are identical proteins named according to their cellular locations. iNAMPT is expressed predominantly in the cytoplasm and nucleus [12], and catalyses the conversion of nicotinamide to nicotinamide mononucleotide, which is a precursor of the cofactor in energy production, NAD^+^. The concentration of NAD^+^ is decreased by ageing, due partly to a reduced iNAMPT level, whereas iNAMPT overexpression increases the lifespan of cells [13]. During high demand for NAD^+^, such as tumorigenesis, NAMPT levels are increased to supply sufficient NAD^+^ to malignant cells [14, 15]. Therefore, NAMPT is a biomarker for certain types of cancer, and iNAMPT inhibitors have been tested as a cancer therapy in clinical trials [16], albeit without success due to toxicity and limited efficacy. In contrast to iNAMPT, eNAMPT is a damage-associated molecular pattern protein that binds to Toll-like receptor 4 (TLR4) to promote innate immunity inflammatory responses [17]. Sources of eNAMPT include adipose tissue and activated inflammatory cells [14]; however, ECs also produce and release abundant eNAMPT under various stress and injury conditions [18, 19]. Increased eNAMPT levels in the circulation are implicated in the severity of cardiovascular diseases such as cardiac ischaemia [20], acute inflammatory lung injury [21] and pulmonary hypertension [22].

People with diabetes and diabetic animal models exhibit increased plasma or serum eNAMPT concentrations [14, 17, 23, 24], and increased eNAMPT levels are an independent risk factor for type 2 diabetes-induced complications [25, 26]. eNAMPT modulates endothelial function, including regulation of vascular integrity [27], vascular tone [28] and endothelial-to-mesenchymal transition [29]. Excess eNAMPT disrupts endothelial barrier function [27, 30] and attenuates endothelium-dependent vascular relaxation [25, 31]. As information on the role of eNAMPT in regulation of coronary endothelial function in diabetes remains extremely limited, the present study is designed to define the role of i/eNAMPT in coronary microvascular dysfunction in diabetes, and to test whether inhibition of i/eNAMPT exhibits therapeutic efficacy in the mitigation of coronary microvascular disease in diabetic mice.

## Methods

### Animals

All experimental protocols used in this study were approved by the Institutional Animal Care and Use Committee (IACUC) at the University of California, San Diego (UCSD), the University of Arizona (UA) and the University of Florida (UF), and conformed to the guidance for the care and use of laboratory animals published by the NIH. The universities have been certified by the US Public Health Service, with Animal Welfare Assurance numbers A3033–01 (UCSD), A3248–01 (UA) and A3377-01 (UF), and IACUC protocol numbers S18185 (UCSD), 14–520 (UA) and 24-001-01 (UF). All laboratory personnel were IACUC-certified.

Inducible type 2 diabetes was induced in male C57BL/6 mice (Envigo, USA) at 6 weeks of age by a single injection of low-dose streptozocin (STZ, 75 mg/kg, dissolved in citrate buffer, i.p.) and a high-fat diet (60% energy from fat; Envigo) for 16 weeks from the day of STZ injection [6, 10, 32]. The experiments were performed 16 weeks after diabetic induction. This is a well-established type 2 diabetes mouse model with hyperglycaemia, hyperinsulinaemia, increased body weight, abnormal glucose tolerance, insulin resistance and dyslipidaemia [6, 10, 32, 33]. Mice only fed with a high-fat diet (without STZ injection) develop obesity but rarely develop type 2 diabetes in our laboratory within 16 weeks. Male TALLYHO/Jng (TH) mice were purchased from the Jackson Laboratory (USA) as a polygenic type 2 diabetes model, with male C57BL/6 mice serving as wild-type (Wt) controls according to the Jackson Laboratory guidelines. Wt and TH mice were fed with a standard laboratory diet (13% energy from fat; Lab Diet, USA) and used for experiments at the age of 16–20 weeks. In addition to inducible C57BL/6 diabetic mice and TH mice, a third mouse line was used, consisting of heterozygous *Nampt*^+/−^ mice on C57BL/6 background as described previously [30]. Homozygous *Nampt* knockout in mice is embryonically lethal [34]. Wt and *Nampt*^+/−^ mice were randomly separated into two groups at 6 weeks old and exposed to the method of type 2 diabetes induction described above. The primer sequence information for genotyping is given in electronic supplementary material (ESM) Table 1. Age was matched (age ± 2 weeks) between diabetic and control mice, and transgenic and Wt mice.

Only male mice were used in this study due to differences in the onset of hyperglycaemia and diabetic complications between male and female mice [35, 36]. It has also been reported that the degree of NAMPT deletion in *Nampt*^+/−^ mice differs between male and female mice [37].

In specific experiments, the NAMPT enzymatic inhibitor FK866 (3 mg/kg) [22, 38] or vehicle (DMSO) was intraperitoneally administered every other day for 4 weeks, starting 12 weeks after diabetes induction. The reported range of effective FK866 doses in mice varies from 0.5 to 100 mg/kg [38, 39], thus the selected dose in this study (3mg/kg) is in the lower range but ultimately proved to be effective in modulating coronary microvascular dysfunction. A humanised eNAMPT-neutralising monoclonal antibody (NAMPT-mAb, ALT-100) [40, 41] was provided by Aqualung Therapeutics (USA). The ALT-100 mAb or human IgG (control) was intraperitoneally administered at 1 mg/kg twice a week for 4 weeks [40].

An oral glucose tolerance test (OGTT) was performed as described previously [6, 10, 32]. After fasting of the mice for 6 h, the glucose levels at baseline were measured (0 min). Mice were then given glucose (2 g/kg body weight) orally, and blood glucose levels were measured at 15, 30 and 60 min after glucose administration. Serum NAMPT concentration was determined using a NAMPT ELISA kit (AdipoGen, USA), and lipid fractions in the plasma were measured using kits from Fujifilm (USA).

Heart dissection was performed under anaesthesia using a mixture of ketamine (100 mg/kg, i.p.) and xylazine (5 mg/kg, i.p.), and all efforts were made to minimise pain during tissue dissection.

### Isolation of mouse CECs

Mouse CECs were isolated using a method described previously [42]. Briefly, after flushing blood from the heart, the heart was dissected, minced and incubated with M199 containing 1 mg/ml collagenase II and 0.6 U/ml dispase II for 1 h at 37°C. The digested material was collected and incubated with magnetic beads prepared by incubation of Dynabeads sheep anti-rat IgG with rat anti-mouse CD31 monoclonal antibody (1 μg/ml) at 4°C overnight. The cell suspension was incubated with beads for 1 h at 4°C, and then CECs were captured and isolated using a Dynal magnet (Thermo Fisher Scientific, USA).

### Western blot analysis

Protein levels were analysed using SDS–PAGE separation and electrophoretic transfer to nitrocellulose membranes. The primary antibodies used in this study are listed in ESM Table 2.

### CFVR measurements

CFVR, rather than coronary flow reserve, was used to assess coronary microvascular function [6] because of the difficulty in precisely measuring coronary arterial diameter in mice [43]. Coronary blood flow velocity (CFV) was measured using a Vevo F2 system (Fujifilm Visual Sonics, Canada). Mice were anaesthetised with isoflurane and kept on a heating pad at 37°C. The resting CFV was obtained at 1% isoflurane. CFVR was defined as the maximal hyperaemic CFV (induced by 2.5% isoflurane) divided by resting CFV (1% isoflurane) (ESM Table 3) [6, 44]. Each experiment was completed within 40 min, and the heart rate was kept above 400 bpm. If the procedure took longer or the heart rate dropped lower than the criteria, the data were eliminated without analysis.

### Assessment of capillary density in the heart

The capillary density in the left ventricle was evaluated as described previously [6]. Briefly, the heart was dissected, embedded in OCT compound, frozen in 2-methylbutane precooled with liquid nitrogen, and kept at −80°C until sectioned. Sections (6 µm in thickness) were fixed in 4% formaldehyde for 5 min, blocked with 5% BSA for 30 min, and incubated with *Bandeiraea simplicifolia* lectin–FITC (BS-l, Sigma Aldrich, USA) for 30 min. BS-l was used to probe the terminal β-galactosyl saccharides associated with ECs on the surface of arterioles, venules and capillaries. The images were captured using a Nikon Eclipse Ti-E microscope (Nikon, Japan) with a 20 × objective lens in a blinded fashion. The capillary count was analysed using ImageJ 1.54 (NIH, USA). The capillary numerical density (number of capillaries per mm^2^) was calculated for each heart.

### Isometric tension measurement in coronary arterial rings

Isometric tension measurement in CAs was performed as described previously [6, 10]. Third-order small CAs were dissected from the hearts and cut into 1 mm segments. The CA rings were mounted on a myograph (DMT, USA) using thin stainless wires (20 μm in diameter), and the resting tension was set at 100 mg. CAs were allowed to equilibrate for 45 min, with washes every 15 min. After equilibration, each CA ring was contracted by treatment with prostaglandin F2α (PGF_2α_) to generate a similar level of contraction in all groups. EDR was assessed by administration of acetylcholine (ACh), and smooth muscle (SM)-dependent relaxation was assessed by administration of sodium nitroprusside (SNP, an NO donor) in a dose-dependent manner (1 nmol/l to 100 μmol/l). EDH-mediated relaxation was assessed by ACh administration in the presence of 100 µmol/l N-Nitro-L-arginine methylester (l-NAME, an eNOS inhibitor) and 10 μmol/l indomethacin (a cyclooxygenase inhibitor that decreases production of prostaglandin I_2_ [PGI_2_]). The degree of relaxation is shown as the percentage of PGF_2α_-induced contraction.

### RNA-seq

mRNA from mouse CECs was isolated using a miRNeasy mini kit (Qiagen, USA), and the RNA samples were sent to the UCSD Institute for Genomic Medicine for bulk RNA-seq. The sequencing libraries were generated using a TruSeq stranded total RNA library preparation kit with rRNA depletion (Illumina, USA). RNA-seq was performed using a NovaSeq 6000 (Illumina), and the data were analysed at the Center for Computational Biology & Bioinformatics at UCSD. We compared the gene expression levels between control mice treated with IgG and diabetic mice treated with IgG, and between diabetic mice treated with IgG and diabetic mice treated with the eNAMPT mAb. Genes with an adjusted *p* value <0.1 were used to generate a Venn diagram. A heatmap was generated showing the genes significantly altered among the three groups that related to angiogenesis and EDR.

### Cell migration assay in human CECs

Human CECs were purchased from commercial suppliers (ESM Table 4) and cultured in EC medium composed of M199 supplemented with 10% FBS, 100 U/ml penicillin, 100 μg/ml streptomycin, 20 μg/ml endothelial cell growth supplement (ECGS), 50 μg/ml d-valine and 16 U/ml heparin. All experiments were performed before passage 10. CECs were plated in a 12-well plate. The next day, the cells were treated with recombinant human wild-type NAMPT (100 ng/ml) or NAD^+^ (100 nmol/l), and the confluent cells were scratched by a pipette tip in the middle of the well. The plate was placed in an EVOS FL auto imaging system (Thermo Fisher Scientific), maintained at 37°C with 20% O_2_, and images were taken every 6 h for 24 h. The percentage cell migration was calculated using ImageJ 1.54, and the data were normalised against the occupied area after 24 h incubation for vehicle-treated cells.

### Measurement of the concentration of mitochondrial ROS

The concentration of mitochondrial reactive oxygen species (ROS) was measured as described previously [10]. Human CECs were stained for 30 min using 5 μmol/l MitoSOX Red (to measure mitochondrial ROS) and 100 nmol/l MitoTracker Green (to visualise the mitochondrial structure). MitoSOX and MitoTracker Green fluorescence from the cells was imaged using a Nikon Eclipse Ti-E fluorescence microscope with a 60 × objective lens. The structure of the mitochondria was determined by the MitoTracker Green signal, and the fluorescence intensity of the MitoSOX in the mitochondria was measured. The background intensity was subtracted from the cell intensity.

### Chemicals

Detailed information on the chemicals used is given in ESM Table 5.

### Scientific rigour and statistics

We established proper control experiments for every experimental plan, and performed data analysis in a blinded fashion wherever possible. The numbers of mice and independent experiments are given in the figure legends. Statistical analysis was performed using GraphPad Prism 10. Data are presented as means ± SEM. If the data passed a normality test (Kolmogorov–Smirnov), two-tailed Student’s *t* test was used to compare two groups, and one-way ANOVA was used for multiple comparisons. If the data did not pass the normality test, a non-parametric test was used (Mann–Whitney for two groups, Kruskal–Wallis for multiple comparisons). Bonferroni’s multiple comparisons test was used as a post hoc test for one-way ANOVA, and Dunn’s test was used as a post hoc test for the Kruskal–Wallis test. A statistical comparison between dose–response curves was performed using two-way ANOVA with a Bonferroni post hoc test. Differences were considered to be statistically significant when the *p* value was <0.05.

## Results

### Serum eNAMPT levels and iNAMPT protein expression in CECs are increased in diabetic mice

**Fig. 1:**
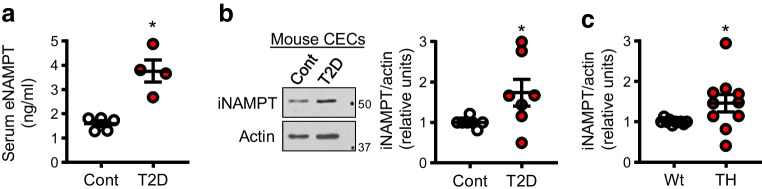
NAMPT levels in diabetic mice. (**a**) Serum eNAMPT levels in control mice (Cont, *n*=5) and inducible type 2 diabetic (T2D) mice (*n*=4). *p<0.05 vs Cont. (**b**) iNAMPT protein levels in CECs isolated from control and T2D mice (*n*=7 per group). *p<0.05 vs Cont. (**c**) iNAMPT protein levels in CECs isolated from Wt and TH mice (*n*=10 per group). *p<0.05 vs Wt. Data are means ± SEM; (**b**, **c**) relative units vs Cont/Wt

We have reported that mice in which type 2 diabetes has been induced by low-dose STZ and high-fat diet feeding have CMD, evidenced by reduced CFVR [6, 32]. Using this model, we first tested eNAMPT levels in the serum and iNAMPT levels in CECs. The serum eNAMPT level in diabetic mice was significantly increased in comparison with control mice (Fig. 1a). In addition, iNAMPT protein levels in CEC lysates were significantly higher in diabetic mice compared with CEC lysates from control mice (Fig. 1b); this was also observed in the spontaneous type 2 diabetes TH mice (Fig. 1c).

### Diabetic *Nampt*^+/−^ mice exhibit improved coronary endothelial function and restored CFVR

**Fig. 2:**
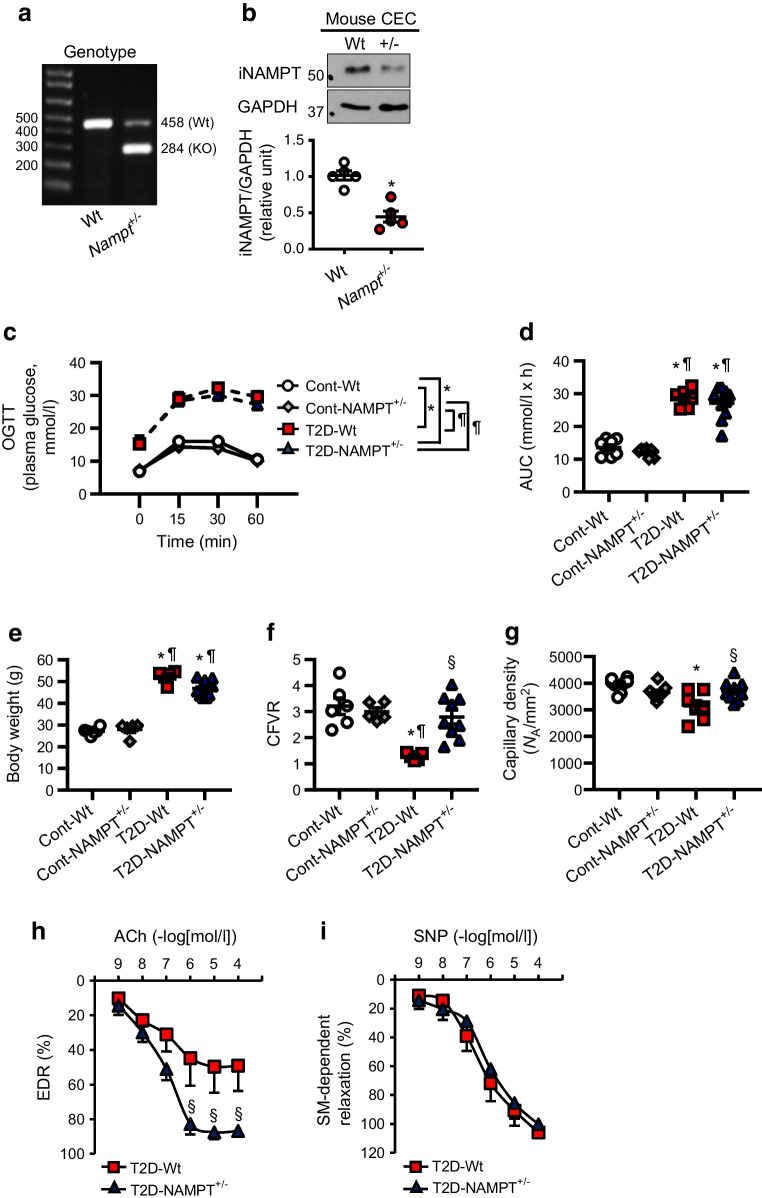
*Nampt*^+/−^ heterozygous knockout mice exhibit improved coronary microvascular function. (**a**) Genotype data obtained using tail samples. (**b**) iNAMPT levels in CECs isolated from Wt and *Nampt*^+/−^ mice (*n*=5 per group). *p<0.05 vs Wt; relative units vs Wt. (**c**) Glucose levels after OGTT and (**d**) AUC in Cont-Wt (*n*=8), Cont-NAMPT^+/−^ (*n*=8), T2D-Wt (*n*=6) and T2D-NAMPT^+/−^ (*n*=10). (**e**) Body weight in Cont-Wt (*n*=6), Cont-NAMPT^+/−^ (*n*=5), T2D-Wt (*n*=5) and T2D-NAMPT^+/−^ (*n*=9). (**f**) CFVR in Cont-Wt (*n*=6), Cont-NAMPT^+/−^ (*n*=5), T2D-Wt (*n*=5) and T2D-NAMPT^+/−^ (*n*=9). (**g**) Capillary density (*N*_A_/mm^2^; number of capillaries per mm^2^) in the left ventricle for Cont-Wt (*n*=8), Cont-NAMPT^+/−^ (*n*=9), T2D-Wt (*n*=7) and T2D-NAMPT^+/−^ (*n*=9). (**h**) EDR in CAs; *n*=4 per group. (**i**) SM-dependent relaxation in CAs; *n*=4 per group. Data are means ± SEM. (**c**–**i**) *p<0.05 vs Cont-Wt; ^¶^*p*<0.05 vs Cont-NAMPT^+/−^; ^§^*p*<0.05 vs T2D-Wt. Cont-Wt, control Wt mice; Cont-NAMPT^+/−^, control *Nampt*^+/−^ mice; T2D-Wt, T2D Wt mice; T2D-NAMPT^+/−^, T2D *Nampt*^+/−^ mice

Homozygous *Nampt* knockout is embryonically lethal; however, heterozygous *Nampt* knockout (*Nampt*^+/−^) mice have exhibited high utility in multiple preclinical studies [22, 30], without visual defects/changes compared with *Nampt*^+/+^ (Wt) mice, but with a significant reduction of iNAMPT levels in CECs (Fig. 2a, b). This reduction of iNAMPT did not affect glucose tolerance (Fig. 2c, d), body weight (Fig. 2e) or lipid profiles (Table 1) in *Nampt*^+/−^ mice with induced diabetes when compared with diabetic Wt mice. Coronary microvascular function determined by CFVR was significantly decreased in diabetic Wt mice compared with control Wt mice, whereas diabetic *Nampt*^+/−^ mice showed a significant increase in CFVR compared with diabetic Wt mice (Fig. 2f), indicating that iNAMPT reduction improves coronary microvascular function in diabetic mice. Reduced CFVR is primarily caused by decreased capillary density in the left ventricle and attenuated vascular relaxation or enhanced vascular contraction in small CAs. Capillary density was significantly reduced in diabetic Wt mice compared with control Wt and control *Nampt*^+/−^ mice, whereas diabetic *Nampt*^+/−^ mice exhibited a significant increase in capillary density (Fig. 2g). We previously reported that EDR is significantly attenuated in inducible diabetic mice compared with the control [6, 10]. Notably, diabetic *Nampt*^+/−^ mice showed a significant increase in EDR (Fig. 2h) with negligible changes to SM-dependent relaxation (Fig. 2i). These data suggest that increased iNAMPT cellular content in diabetic mice leads to coronary endothelial dysfunction and attenuates coronary microvascular function.

**Table 1:** Lipid profiles

	TC (mmol/l)	HDL (mmol/l)	TG (mmol/l)
iNAMPT/eNAMPT inhibition			
Wt control (*n*=8)	1.58 ± 0.20	0.65 ± 0.08	0.21 ± 0.03
*Nampt*^+/−^ control (*n*=9)	1.40 ± 0.16	0.57 ± 0.07	0.18 ± 0.03
T2D Wt (*n*=6)	4.63 ± 0.37^*,†^	2.34 ± 0.21^*,†^	0.47 ± 0.07^*,†^
T2D *Nampt*^+/−^ (*n*=9)	5.49 ± 0.33^*,†^	2.62 ± 0.19^*,†^	0.45 ± 0.05^*,†^
iNAMPT inhibition			
Control + vehicle (*n*=10)	2.54 ± 0.10	1.51 ± 0.07	0.29 ± 0.08
T2D + vehicle (*n*=10)	5.23 ± 0.12^§^	2.75 ± 0.07^§^	0.27 ± 0.08
T2D + FK866 (*n*=10)	4.64 ± 0.37^§^	2.94 ± 0.47^§^	0.33 ± 0.08
eNAMPT inhibition			
Control + IgG (*n*=8)	2.09 ± 0.18	1.40 ± 0.08	0.40 ± 0.09
T2D + IgG (*n*=8)	5.88 ± 0.50^¶^	2.71 ± 0.16^¶^	0.62 ± 0.07^¶^
T2D + NMAPT mAb (*n*=8)	6.80 ± 0.40^¶^	2.99 ± 0.18^¶^	0.67 ± 0.06^¶^

Data are means ± SEM

*n* indicates the number of mice

^*^p<0.05 vs Wt control; ^†^*p*<0.05 vs *Nampt*^+/−^ control; ^§^*p*<0.05 vs control + vehicle; ^¶^*p*<0.05 vs control + IgG

HDL, plasma HDL; T2D, type 2 diabetes; TC, plasma total cholesterol; TG, plasma triglycerides

### i/eNAMPT inhibition improves CMD in diabetic mice

**Fig. 3:**
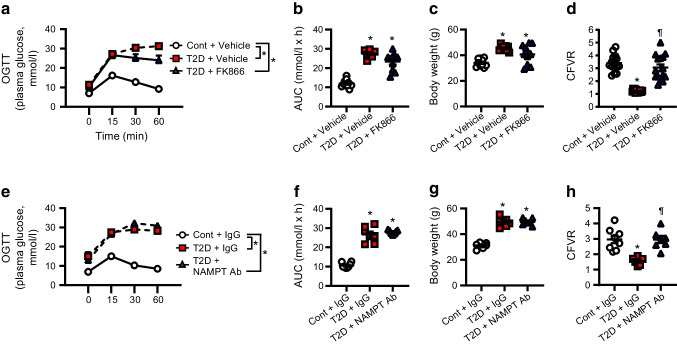
NAMPT inhibition restored coronary microvascular function. (**a**, **b**) Effect of FK866 on glucose levels after OGTT (**a**) and AUC (**b**) in control mice treated with vehicle (Cont + Vehicle; *n*=10), T2D mice treated with vehicle (T2D + Vehicle; *n*=9) and T2D mice treated with FK866 (T2D + FK866; *n*=10). (**c**) Body weight in Cont + Vehicle mice (*n*=10), T2D + Vehicle mice (*n*=9) and T2D + FK866 mice (*n*=10). (**d**) CFVR in Cont + Vehicle mice (*n*=15), T2D + Vehicle mice (*n*=14) and T2D + FK866 mice (*n*=15). Data are means ± SEM. *p<0.05 vs Cont + Vehicle; ^¶^*p*<0.05 vs T2D + Vehicle. (**e**, **f**) Effect of eNAMPT mAb on glucose levels after OGTT (**e**) and AUC (**f**) in control mice treated with IgG (Cont + IgG; *n*=8), T2D mice treated with IgG (T2D + IgG; *n*=7) and T2D mice treated with eNAMPT mAb (T2D + NAMPT Ab; *n*=7). (**g**) Body weight in Cont + IgG mice (*n*=8), T2D + IgG mice (*n*=7) and T2D + NAMPT Ab mice (*n*=7). (**h**) CFVR in Cont + IgG mice (*n*=8), T2D + IgG mice (*n*=6) and T2D + NAMPT Ab mice (*n*=7). Data are means ± SEM. *p<0.05 vs Cont + IgG; ^¶^*p*<0.05 vs T2D + IgG

Chronic administration of the cell-permeable and selective NAMPT inhibitor FK866 did not alter the glucose tolerance, body weight or lipid profile in diabetic mice (Fig. 3a–c and Table 1). However, consistent with results in *Nampt*^+/−^ mice, FK866 administration in diabetic mice significantly improved CFVR (Fig. 3d). Next, we examined the role of eNAMPT in the development of coronary microvascular dysfunction in diabetes. eNAMPT was inhibited by 4 weeks of treatment with eNAMPT-neutralising mAb in diabetic mice. In line with the results from NAMPT inhibition by FK866 and genetic NAMPT reduction in *Nampt*^+/−^ mice, administration of eNAMPT mAb did not result in alterations to glucose tolerance, body weight or lipid profiles in diabetic mice (Fig. 3e–g and Table 1), but restored coronary microvascular function in diabetes, as evidenced by restoration of the CFVR (Fig. 3h). These data suggest that both iNAMPT and eNAMPT contribute to the development of CMD in diabetes, and that inhibition of either iNAMPT or eNAMPT is sufficient to restore coronary microvascular function in diabetic mice.

### eNAMPT mAb administration in diabetic mice restores expression levels of mRNAs related to angiogenesis and EDR in CECs

**Fig. 4:**
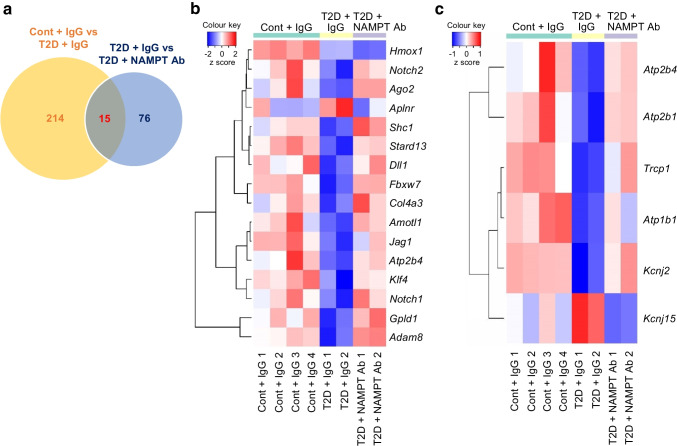
eNAMPT mAb administration in diabetic mice ameliorates mRNA levels for genes related to angiogenesis and EDR. (**a**) Venn diagram showing the number of significant DEGs in mouse CECs between control mice treated with IgG (Cont + IgG; *n*=4), T2D mice treated with IgG (T2D + IgG; *n*=2) and T2D mice treated with eNAMPT mAb (T2D + NAMPT Ab; *n*=2). (**b**) Heatmap showing significant DEGs related to angiogenesis. (**c**) Heatmap showing significant DEGs related to EDR. The Venn diagram and heatmaps were generated using genes with adjusted *p* values <0.1

RNA-seq data in isolated CECs revealed 229 significantly differentially expressed genes (DEGs) between control and diabetic mice (diabetic DEGs) and 91 DEGs that were affected by eNAMPT mAb administration in diabetic mice (eNAMPT mAb DEGs), with a total of 15 DEGs being common to both DEG datasets (Fig. 4a). Gene ontology analysis revealed that the diabetic DEGs were related to angiogenesis (Fig. 4b) and Ca^2+^/K^+^ handling (Fig. 4c), with expression of these DEGs being rectified in diabetic mice receiving the eNAMPT mAb.

### NAMPT and NAD^+^ treatments decrease migration of CECs

**Fig. 5:**
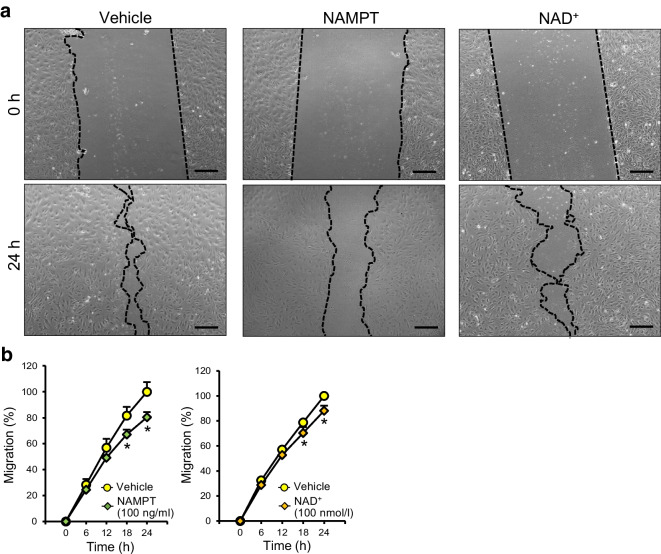
eNAMPT and NAD^+^ attenuate endothelial migration. (**a**) Representative photomicrographs of cell migration in human CECs treated with vehicle, eNAMPT (100 mg/ml) or NAD^+^ (100 nmol/l). Scale bars, 200 μm. (**b**) Summarised cell migration data. The data were normalised by the average cell migration percentage in vehicle-treated CECs at 24 h. Data are presented as mean ± SEM for six experiments per group. *p<0.05 vs vehicle

Capillary density in tissues is regulated by the balance of EC migration, EC apoptosis and incorporation of endothelial progenitor cells into the injured area. Therefore, the next set of experiments was designed to test the effect of NAMPT and NAD^+^ on endothelial migration. Figure 5 shows that eNAMPT and NAD^+^ slightly, but significantly, attenuated endothelial migration, implying that the reduced capillary density seen in diabetes may be due partly to attenuated endothelial migration caused by increased eNAMPT levels.

### eNAMPT and NAD^+^ treatments attenuate EDR via increasing mitochondrial ROS formation in CECs

**Fig. 6:**
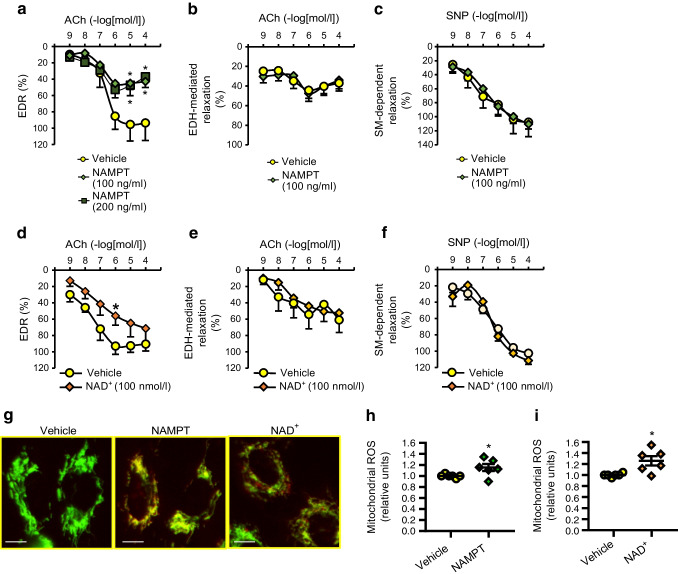
eNAMPT and NAD^+^ reduce endothelial-dependent relaxation in CAs via increased mitochondrial ROS formation in CECs. (**a**) EDR in the presence or absence of 100 or 200 ng/ml eNAMPT; *n*_CAs_ = 6 per group. (**b**) EDH-mediated relaxation, assessed by ACh administration in the presence of l-NAME (an eNOS inhibitor, 100 µmol/l) and indomethacin (a cyclo-oxygenase inhibitor, 10 µmol/l), with or without NAMPT (100 ng/ml). Vehicle, *n*_CAs_ = 11; eNAMPT, *n*_CAs_ = 9. (**c**) SM-dependent relaxation in the presence or absence of eNAMPT; *n*_CAs_ = 6 per group. (**d**) EDR in the presence or absence of NAD^+^ (100 nmol/l); *n*_CAs_ = 5 per group. (**e**) EDH-mediated relaxation in CAs with or without NAD^+^, assessed by ACh administration in the presence of l-NAME and indomethacin; *n*_CAs_ = 4 per group. (**f**) SM-dependent relaxation in the presence or absence of NAD^+^; *n*_CAs_ = 7 per group. (**g**) Photomicrographs of mitochondrial ROS in CECs treated with vehicle, eNAMPT (100 mg/ml) or NAD^+^ (100 nmol/l). Scale bars, 10 μm. (**h**) Summarised data showing the average mitochondrial ROS intensity in vehicle- and eNAMPT-treated CECs for six experiments with >110 cells per group. (**i**) Summarised data showing the average mitochondrial ROS intensity in vehicle- and NAD^+^-treated CECs for six experiments with >120 cells. Data are means ± SEM; (**h**, **i**) relative units vs vehicle. *p<0.05 vs vehicle

A 1 h treatment with eNAMPT or NAD^+^ significantly attenuated EDR (Fig. 6a, d) but had minimal effects on either EDH-mediated relaxation (Fig. 6b, e) or SM-dependent relaxation (Fig. 6c, f). To address the hypothesis that NAMPT or NAD^+^ may alter NO bioavailability in ECs, we measured mitochondrial ROS formation in CECs using mitoSOX. The results showed that eNAMPT or NAD^+^ treatment significantly increased mitochondrial ROS production in CECs (Fig. 6g–i), suggesting that eNAMPT or NAD^+^ attenuate EDR due partly to increased ROS generation.

## Discussion

In addition to CAD, CMD is a significant risk factor for cardiac ischaemia. However, the development of specific CMD treatments lags far behind treatments for CAD due to the lack of informative preclinical CMD models. We show that our diabetic mouse model is an ideal animal model to investigate the mechanisms of CMD induced by diabetes as this mouse model exhibits reduced CFVR without atherosclerotic plaque formation, and displays capillary rarefaction in the left ventricle and attenuated vascular relaxation in small CAs (the leading causes of CMD) [6, 32]. The present study provides compelling support for eNAMPT inhibition as a novel and effective therapeutic strategy for CMD in diabetes.

Diabetic individuals show increased circulating eNAMPT levels [14, 23, 24, 45] as well as increased iNAMPT levels in adipose tissues [46] and mononuclear cells [47]. Goktas et al demonstrated that liver iNAMPT concentrations positively correlate with HOMA-IR values [46]. However, whether i/eNAMPT participates in glucose homeostasis during diabetes remains unclear [45]. We used three methods to alter i/eNAMPT function/availability in diabetic mice, and examined their glucose tolerance, body weight and lipid profiles. Our data show that i/eNAMPT are unlikely to regulate glucose homeostasis and dyslipidaemia in diabetic mice (Figs 2c–e, 3a–c, e–g and Table 1). These data also imply that changes in endothelial function after NAMPT inhibition are not caused by alteration in diabetic status. NAMPT is essential in fundamental physiological functions; however, a pathophysiological increase of NAMPT levels is implicated in the development of many cardiovascular diseases [14, 22, 30]. Diabetic mice show increased eNAMPT levels (Fig. 1a), in agreement with a previous study [17]. As adipose tissue is an important source of eNAMPT [14], the increased body weight in diabetic mice (Fig. 2e) due to fat accumulation may be responsible for increased serum eNAMPT. We also found that iNAMPT levels are increased in CECs from diabetic mice (Fig. 1b, c), which may contribute to the elevated serum eNAMPT levels observed in these mice. Whether the increased eNAMPT levels observed in diabetic humans or preclinical models reflect orchestrated cellular secretion or pyroptotic release via membrane rupture is still unknown.

To our knowledge, we are the first to report that i/eNAMPT inhibition restores coronary microvascular function via improving coronary endothelial function (Fig. 2). Capillary density is regulated by proliferation and migration of mature ECs, endothelial apoptosis, and incorporation of endothelial progenitor cells into the site where ECs are lost or injured. In diabetes, coronary endothelial angiogenesis and migration are attenuated [32] and EC apoptosis in the heart is increased [6, 32]. The reduced NAMPT expression in *Nampt*^+/−^ mice significantly increased capillary density in diabetic mice (Fig. 2g). eNAMPT mAb administration in diabetic mice also restored mRNA expression levels of genes related to angiogenesis (Fig. 4), and ex vivo data showed that eNAMPT and NAD^+^ treatment in CECs attenuate EC migration (Fig. 5). These observations suggest that increased serum eNAMPT and eNAMPT/TLR4 signalling attenuate EC migration in the heart, resulting in reduced capillary density in diabetic mice. During tumorigenesis, i/eNAMPT induce cell proliferation and migration [48, 49]. Therefore, it was a total surprise for us to observe that NAMPT attenuates EC migration. Studies have observed the heterogeneity of cells among different tissues [15], while endothelial moiety and function differ significantly among ECs from different organs [50, 51]. Therefore, this unique response of CECs to NAMPT (or the inhibitory effect of NAMPT on CEC migration) requires further investigation to define its mechanisms.

Diabetic *Nampt*^+/−^ mice showed a significant increase in EDR compared with diabetic Wt mice (Fig. 2h). Furthermore, eNAMPT and NAD^+^ treatment in CAs significantly attenuated EDR (Fig. 6a, d) without affecting EDH-mediated relaxation (Fig. 6b, e). ACh stimulates the production of NO and prostacyclin and induces membrane hyperpolarisation in ECs. NO, prostacyclin and hyperpolarised potential are then diffused to SM cells and cause vasodilation; this signalling cascade is termed EDR. In the 3rd order of CAs, we observed significant endothelium-derived NO- and EDH-dependent relaxation in control mice, whereas diabetic mice showed attenuated endothelium-derived NO- and EDH-mediated relaxation [6, 10, 11]. As EDH-mediated relaxation was not altered by eNAMPT or NAD^+^ treatment (Fig. 6b, e), the attenuation of EDR by eNAMPT or NAD^+^ may be due to reduced endothelium-derived NO-dependent relaxation. NO bioavailability is regulated by NO production and NO degradation. NO production is controlled by eNOS activity, which is Ca^2+^-dependent [11]. Therefore, the change in expression of genes related to ion channels/pumps by eNAMPT mAb treatment (Fig. 4c) may contribute to restoring endothelium-derived NO-dependent relaxation. We also demonstrate that eNAMPT and NAD^+^ increase mitochondrial ROS formation, maybe via eNAMPT/TLR4 inflammatory cascade signalling (Fig. 6g, i). Thus, excessive production of mitochondrial ROS elicited by eNAMPT/TLR4 or NAD^+^ may play a critical role in reducing NO bioavailability and attenuating EDR in CAs in people with diabetes. We previously reported that CECs in diabetic mice produced excess mitochondrial ROS, and that chronic administration of mitoTempol (a mitochondrial ROS scavenger) restored the attenuated EDR in CAs of diabetic mice [10]. In patients with type 2 diabetes, flow-mediated vasodilation (a non-invasive assessment of endothelial function) negatively correlates with plasma eNAMPT concentration [25, 52]. Romacho et al showed that eNAMPT administration attenuates EDR in the mesenteric artery, and that this is restored by treatment with FK866 or TLR4 blocker, implying that increased NAD^+^ and activation of inflammatory signalling by eNAMPT/TLR4 activation contribute to impairment of EDR by eNAMPT [31]. Vallejo et al also demonstrated that eNAMPT attenuates EDR via overproduction of cytosolic ROS [53]. TLR4 activation has been implicated in attenuated EDR via excess ROS production in ageing mice [54] and spontaneous hypertensive rats [55], or due to reduced eNOS activation in *db*/*db* mice [56]. Taken together, these data imply that secretion of eNAMPT, a potent damage-associated molecular pattern and TLR4 ligand, attenuates EDR, which may subsequently lead to the coronary microvascular dysfunction observed in diabetes.

In this study, we used FK866 as an iNAMPT inhibitor, eNAMPT-neutralising mAb as an eNAMPT inhibitor, and *Nampt* heterozygous knockout mice as an iNAMPT/eNAMPT-inhibited mouse model. FK866 inhibits the enzymatic activity of NAMPT and thus inhibits both iNAMPT and eNAMPT. However, the relevance of the extracellular enzymatic activity of eNAMPT is still uncertain, as decreases in serum nicotinamide mononucleotide (NMN) were never observed after FK866 administration. In addition, there is inconsistent evidence as to whether eNAMPT generates NMN extracellularly and influences cellular function. Whereas the FK866 inhibitor is cell-permeable and effectively inhibits iNAMPT enzymatic activity, the eNAMPT mAb is delivered intraperitoneally and does not access the intracellular compartment to influence intracellular NAD levels.

iNAMPT plays a critical role in maintaining the physiological function of cells by generating NAD^+^; however, NAD^+^ application to control ECs or control vessels significantly attenuated endothelial function in our study (Figs 5 and 6), implying that a pathophysiological increase in iNAMPT has a maladaptive effect on endothelial function. Therefore, inhibition of iNAMPT by FK866 showed a beneficial effect on coronary microvascular function in diabetic mice in which iNAMPT is overexpressed above its physiological level.

Our studies using the humanised eNAMPT-neutralising ALT-100 mAb, which is currently in phase 2A clinical trials (ClinicalTrials.gov, NCT05938036), highlight the critical role of circulating eNAMPT as a damage-associated molecular pattern [17]. The ALT-100 mAb has been shown to reduce the severity of diverse preclinical models of non-alcoholic fatty liver disease (NASH)-induced hepatic fibrosis [17], radiation-induced lung fibrosis [57], ischaemia-induced cardiac fibrosis [20] and pulmonary hypertension [22] underscoring the importance of our current findings in diabetic mice with CMD.

In conclusion, we present compelling data indicating that diabetes leads to increased iNAMPT expression in CECs and augmented eNAMPT secretion that subsequently attenuates CEC migration, inhibits EDR in CAs and impairs coronary microvascular function. Our highly translational studies, using global *Nampt*^+/−^ heterozygous knockout mice, an iNAMPT enzymatic inhibitor and an eNAMPT-neutralising mAb, indicate that NAMPT is a promising therapeutic target for developing novel and unique treatments for CMD in people with diabetes.

## Supplementary Information

Below is the link to the electronic supplementary material.

ESM 1 (PDF 272 KB)

## Data Availability

All data in the current study are available from the corresponding author upon reasonable request.

## Abbreviations

ACh: Acetylcholine
BS-l: *Bandeiraea simplicifolia* lectin–FITC
CA: Coronary artery
CAD: Coronary artery disease
CEC: Cardiac endothelial cell
CFV: Coronary blood flow velocity
CFVR: Coronary flow velocity reserve
CMD: Coronary microvascular disease
ECs: Endothelial cells
EDH: Endothelium-dependent hyperpolarisation
EDR: Endothelium-dependent relaxation
eNAMPT: Extracellular nicotinamide phosphoribosyltransferase
iNAMPT: Intracellular nicotinamide phosphoribosyltransferase
*Nampt*^+/−^: Heterozygous *Nampt*
ROS: Reactive oxygen species
SM: Smooth muscle
SNP: Sodium nitroprusside
STZ: Streptozocin
TH: TALLYHO/Jng
TLR4: Toll-like receptor 4
Wt: Wild-type
