# Supplementary material for: eNAMPT is a novel therapeutic target for mitigation of coronary microvascular disease in type 2 diabetes
Source: Diabetologia. 2024 Jun 19;67(9):1998–2011. doi: 10.1007/s00125-024-06201-9 (PMC11410976; doi:10.1007/s00125-024-06201-9)
Supplement: Supplementary file 1 — ESM 1 (PDF 272 KB) [file 125_2024_6201_MOESM1_ESM.pdf]

Supplemental materials

ESM Table 1. Primers used for genotyping

Strain	Forward	Reverse
NAMPT Wt/KO	5'-CAGCAGCAGACCATTTTCAA-3'	
NAMPT Wt		5'-CGGATGCCTTAGCCTGAAGT-3'
NAMPT KO		5' -GGGAGTGACACACAGCAAATCA-3'

ESM Table 2. Antibodies

Target antigen	Company	Catalog #	Host	Dilution Rate	Buffer
Actin	Santa Cruz Biotechnology Inc (Dallas,TX)	sc-1616	Goat	1:4000	1% BSA in TBSt
NAMPT	Santa Cruz Biotechnology Inc (Dallas,TX)	sc-393510	Mouse	1:4000	1% BSA in TBSt
GAPDH	Thermo-Fisher Scientific (Waltham, MA)	MA5-15738	Mouse	1:5000	1% BSA in TBSt
Anti-goat					
Anti-mouse					

The antibody for NAMPT Ab was validated using the cell lysate from a NAMPT$^{+/-}$ knockout mouse. Actin and GAPDH were used as loading controls.

ESM Table 3. Coronary flow velocity at the resting and peak

	Resting (mm/s)	Peak (mm/s)
Cont-Wt	420.5 ± 51.7	1328.6 ± 169.9
Cont-NAMPT$^{+/-}$	497.7 ± 59.7	1507.6 ± 242.8
T2D-Wt	570.4 ± 87.1	739.5 ± 124.5¶
T2D-NAMPT$^{+/-}$	324.7 ± 29.4§	867.0 ±70.1¶
Cont + Vehicle	488.7 ± 42.0	1616.8 ± 103.9*
T2D + Vehicle	784.9 ± 124.3*	945.2 ± 137.1
T2D + FK866	556.8 ± 53.1	1654.1 ± 156.7¶
Cont + IgG	507.9 ± 57.9	1422.8 ± 109.5
T2D + IgG	624.8 ± 59.7	947.8 ± 57.1*
T2D + NAMPT Ab	430.9 ± 29.9	1237.9 ± 55.5

NAMPT$^{+/-}$ groups: Cont-Wt, *n*=6; Cont-NAMPT$^{+/-}$, *n*=5; T2D-Wt, *n*=5; T2D- NAMPT$^{+/-}$ *n*=9. **p*<0.05 vs. Cont-Wt, ¶*p*<0.05 vs. Cont-NAMPT$^{+/-}$, §*p*<0.05 vs. T2D-Wt. FK866 groups: *n*=15; T2D + Vehicle, *n*=14; T2D + FK866, *n*=15. **p*<0.05 vs. Cont + Vehicle, ¶*p*<0.05 vs. T2D + Vehicle. NAMPT Ab groups: Cont + IgG, *n*=8; T2D + IgG, *n*=6; T2D + eNAMPT mAb, *n*=7. **p*<0.05 vs. Cont + IgG. Data are presented as means ± SEM.

ESM Table 4. Cultured cells

Name	Company	Catalog #	Lot#	Sex (F, M)	Diagnosis
HCEC	Lonza (Basel, Switzerland)	cc-2585	305907	M	Control
HCEC	Lonza (Basel, Switzerland)	cc-2585	547320	M	Control
HCEC	Lonza (Basel, Switzerland)	cc-2585	7F4281	M	Control
HCEC	Cell Applications Inc. (CA, USA)	300-05a	2991	M	Control

The authentication and mycoplasma contamination status of the cell lines are attached on the last page of this file.

ESM Table 5. Chemicals

Description	Company	Catalog #
Acetylcholine	Sigma Aldrich, (MO, USA)	A9101
Collagenase II	Worthington Biochemical Corp. (NJ, USA)	LS004176
Diet (normal diet, 13% kcal from fat)	Lab Diet (MO, USA)	5001
Diet (high fat diet, 60% kcal from fat)	Envigo RMS Inc. (IN, USA)	TD.06414
Dispase II	Worthington Biochemical Corp. (NJ, USA)	LS02109
Dynabeads ® Sheep Anti-Rat IgG	Thermo-Fisher Scientific, (Waltham, MA)	11035
Endothelial Cell Growth Supplement	Thermo-Fisher Scientific, (Waltham, MA)	356006
Fetal Bovine Serum	Thermo-Fisher Scientific, (Waltham, MA)	MT35010CV
FK866	Sigma Aldrich, (MO, USA)	F8557
Human IgG	Jackson ImmunoResearch Labs (West Grove, PA)	209-005-088
indomethacin	Sigma Aldrich, (MO, USA)	I7378
Iron-Supplemented Calf Serum	Thermo-Fisher Scientific, (Waltham, MA)	SH30072.04
Isoflurane	Henry Schein(NY, USA)	1169567762
Ketamine	Henry Schein (NY, USA)	VINB-KET0-7021
Lectin-FITC	Sigma Aldrich (MO, USA)	L9381
L-NAME	Cayman Chemical	80210
Lucifer Yellow CH, Lithium Salt,	Thermo-Fisher Scientific, (Waltham, MA)	L453
MitoSOX™	Thermo-Fisher Scientific, (Waltham, MA)	M36008
Medium 199	Thermo-Fisher Scientific, (Waltham, MA)	MT10060-CV
miRNeasy Mini Kit	Qiagen (CA, USA)	217004
NAD^+	Sigma Aldrich, (MO, USA)	N8285
NAMPT ELISA Kit	AdipoGen Corp. (CA, USA)	AG-45A-0007YEK-KI01
NAMPT antibody (immune-based humanized monoclonal)	Aqualung Therapeutics (AZ, USA)	AT-100
NAMPT recombinant protein (Human)	MBL International (Woburn, MA)	CY-E1251
$PGF_{2\alpha}$	Sigma Aldrich (MO, USA)	P0424-5 mg
Rat anti-mouse CD31	BD Biosciences (CA, USA)	553370
Sodium Nitroprusside	Sigma Aldrich, (MO, USA)	71778
Streptomycin/penicillin	Thermo-Fisher Scientific, (Waltham, MA)	MT-30-002-CI
Streptozotocin (STZ)	VWR (PA, USA)	89149-800
Trypsin/EDTA	Thermo-Fisher Scientific, (Waltham, MA)	MT25052-CI
Xylazine	Henry Schein (NY, USA)	NADA # 139-236
Other general chemicals	Sigma Aldrich, (MO, USA)	

Lonza Walkersville Inc.
8830 Biggs Ford Road
Walkersville, MD 21793 8415
[illegible] (301) 898 7025
[illegible]x (301) 845 4024

control.
ordered 1/8/15.

Lonza

Printed on, 19-Jun-2015 14:56

CERTIFICATE OF ANALYSIS

Product Code: CC-2585
Product: HCAEC-Coronary Art. Endo Cells, EGM-2MV, cryo amp

Lot Number: 0000305907
Manufacture Date: 15-Jun-2012

TEST (Method)	SPECIFICATIONS Min.	Max.	Results
Tissue Acquisition Number	***	***	24493
DONOR CHARACTERISTICS			
Age	***	***	27 Y
Sex	***	***	MALE
Race	***	***	H/B
VIRUS TESTING			
[illegible]IV Test	***	***	Not detected
[illegible]V Test	***	***	Not detected
HCV Test	***	***	Not detected
MICROBIAL TESTING			
Sterility Test	***	***	Negative
Mycoplasma	***	***	Negative
CELL PERFORMANCE TESTING			
Cell Passage Frozen			3
Viability	>= 70%	***	92 %
Cell Count (Cells/ml)	>= 500,000	***	555000
Seeding Efficiency	>= 20%	***	91 %
Doubling Time (hours)	15	48	25 hrs
Alpha Actin Expression	Pass: Negative	***	Pass

These cells were isolated from donated human tissue after obtaining permission for their use in research applications by informed consent or legal authorization. This product is for research use only. Details concerning the use of our cell and media products can be downloaded from our website at www.lonza.com/cell-protocols.

In addition to the specifications listed above, the following are guaranteed for all lots of this product using Lonza's Clonetics (TM) and Poietics (TM) Media, Reagents, and Protocols: Total Population Doublings >=15, Acetylated LDL Uptake Positive, Factor VIII Positive.

[illegible]is lot has been reviewed by Quality Assurance in compliance with requirements of Lonza's Quality System.
[illegible]his document was generated from a validated Part 11-compliant electronic system and thus handwritten signatures are not required.

Lonza Walkersville Inc.
8830 Biggs Ford Road
Walkersville, MD 21793 8415
Tel (301) 898 7025
Fax (301) 845 4024

Lonza

CERTIFICATE OF ANALYSIS

Product Code: CC-2585
Product: HCAEC-Coronary Art. Endo Cells, EGM-2MV, cryo amp

Lot Number: 7F4281
Manufacture Date: 12-Nov-2007

TEST (Method)	SPECIFICATIONS Min.	Max.	Results
Tissue Acquisition Number	***	***	16339-1
DONOR CHARACTERISTICS			
Age	***	***	21 Y
Sex	***	***	MALE
Race	***	***	C
VIRUS TESTING			
HIV Test	***	***	Not detected
HBV Test	***	***	Not detected
HCV Test	***	***	Not detected
MICROBIAL TESTING			
Sterility - Amp	***	***	Negative
Direct Plating (Mycoplasma)	***	***	Negative
CELL PERFORMANCE TESTING			
Cell Passage Frozen			3
Viability	>= 70%	***	83 %
Cell Count (Cells/ml)	>= 500,000	***	716000
Seeding Efficiency	>= 20%	***	64 %
Doubling Time (hours)	15	48	20 hrs
Alpha Actin Expression	NEGATIVE	***	Pass

These cells were isolated from donated human tissue after obtaining permission for their use in research applications by informed consent or legal authorization. This product is for research use only. Details concerning the use of our cell and media products can be downloaded from our website at www.lonza.com/cell-protocols.

In addition to the specifications listed above, the following are guaranteed for all lots of this product using Lonza's Clonetics (TM) and Poietics (TM) Media, Reagents, and Protocols: Total Population Doublings >=15, Acetylated LDL Uptake Positive, Factor VIII Positive.

This lot has been reviewed by Quality Assurance in compliance with requirements of Lonza's Quality System.
This document was generated from a validated Part 11-compliant electronic system and thus handwritten signatures are not required.

Lonza Walkersville Inc.
8830 Biggs Ford Road
Walkersville, MD 21793 8415
Tel (301) 898 7025
Fax (301) 845 4024

Lonza

CERTIFICATE OF ANALYSIS

**Product Code:**	CC-2585	**Lot Number:**	0000547320
**Product:**	HCAEC-Coronary Art. Endo Cells, EGM-2MV, cryo amp	**Manufacture Date:**	13-May-2016

TEST (Method)	SPECIFICATIONS Min.	Max.	Results
Tissue Acquisition Number	***	***	29790
DONOR CHARACTERISTICS			
Age	***	***	55 Y
Sex	***	***	MALE
Race	***	***	B
VIRUS TESTING			
HIV Test	***	***	Not Detected
HBV Test	***	***	Not Detected
HCV Test	***	***	Not Detected
MICROBIAL TESTING			
Sterility Test	***	***	Negative
Mycoplasma	***	***	Negative
CELL PERFORMANCE TESTING			
Cell Passage Frozen			3
Viability	>= 70%	***	95 %
Cell Count (Cells/ml)	>= 500,000	***	1115000
Seeding Efficiency	>= 20%	***	23 %
Doubling Time (hours)	15	48	22 hrs
Alpha Actin Expression	Pass: Negative	***	Pass

These cells were isolated from donated human tissue after obtaining permission for their use in research applications by informed consent or legal authorization. This product is for research use only. Details concerning the use of our cell and media products can be downloaded from our website at www.lonza.com/cell-protocols.

In addition to the specifications listed above, the following are guaranteed for all lots of this product using Lonza's Clonetics (TM) and Poietics (TM) Media, Reagents, and Protocols: Total Population Doublings >=15, Acetylated LDL Uptake Positive, Factor VIII Positive.

This lot has been reviewed by Quality Assurance in compliance with requirements of Lonza's Quality System.
This document was generated from a validated Part 11-compliant electronic system and thus handwritten signatures are not required.

CELL
APPLICATIONS, INC.

RcVD 10/3/17
thawed 10/7/17 P2

CELL
APPLICATIONS, INC.
Cat 300-05a Lot 2991
HCAEC
Human Coronary Artery Endothelial Cells
For research use only Store in liquid nitrogen
858-453-0848 ♦ www.cellapplications.com

PRODUCT SPECIFICATION SHEET

CRYOPRESERVED HCAEC

Human Coronary Artery Endothelial Cells

HCAEC (Human Coronary Artery Endothelial Cells) are primary normal human endothelial cells isolated from healthy coronary arteries. HCAEC were cultured in Endothelial Cell Growth Medium and cryopreserved at second passage. HCAEC can be cultured and propagated to 5th passage and beyond without losing their morphologic and phenotypic characteristics.

Catalog No.: 300-05a

Lot No.: 2991

Quantity: ~500,000 cells in an ampoule

Source: Normal human coronary arteries. Single donor: 27 years Caucasian male.

Formulation: Cryopreserved at the second passage in Endothelial Cell Basal Medium containing 10% FBS and 10% DMSO.

Quality Control:

Cell Viability: 94.1%
Doubling Time: 36.5 hours.
Population Doubling: 15
Characterization: Factor VIII-related antigen expression: Positive
DiI-Ac-LDL uptake: Positive
Bioassay: Each lot is tested for its:
--Ability to attach and spread on tissue culture ware surface, and proliferate in Endothelial Cell Growth Medium.

Potential Biological Contaminants

Agents	Test Method	Results
HIV-1 virus	Oligonucleotide-directed amplifications	Not detected
Hepatitis B virus	Oligonucleotide-directed amplifications	Not detected
Hepatitis C virus	Oligonucleotide-directed amplifications	Not detected
Mycoplasma	Oligonucleotide-directed amplifications	Not detected
Bacteria, yeast, fungi	Long term antibiotic-, antimycotic-free culture	Not detected

Storage & Stability: Stable when stored properly in liquid nitrogen.
